# Supplementary material for: Paninvasion severity assessment of a U.S. grape pest to disrupt the global wine market
Source: Commun Biol. 2022 Jul 4;5:655. doi: 10.1038/s42003-022-03580-w (PMC9253006; doi:10.1038/s42003-022-03580-w)
Supplement: Supplementary file 2 — Reporting Summary [file 42003_2022_3580_MOESM2_ESM.pdf]

# nature portfolio

Corresponding author(s): Nicholas A. Huron

Last updated by author(s): May 5, 2022

# Reporting Summary

Nature Portfolio wishes to improve the reproducibility of the work that we publish. This form provides structure for consistency and transparency in reporting. For further information on Nature Portfolio policies, see our Editorial Policies and the Editorial Policy Checklist.

## Statistics

For all statistical analyses, confirm that the following items are present in the figure legend, table legend, main text, or Methods section.

| n/a | Confirmed | |
|---|---|---|
| ☐ | ☒ | The exact sample size ($n$) for each experimental group/condition, given as a discrete number and unit of measurement |
| ☒ | ☐ | A statement on whether measurements were taken from distinct samples or whether the same sample was measured repeatedly |
| ☐ | ☒ | The statistical test(s) used AND whether they are one- or two-sided<br>*Only common tests should be described solely by name; describe more complex techniques in the Methods section.* |
| ☐ | ☒ | A description of all covariates tested |
| ☐ | ☒ | A description of any assumptions or corrections, such as tests of normality and adjustment for multiple comparisons |
| ☐ | ☒ | A full description of the statistical parameters including central tendency (e.g. means) or other basic estimates (e.g. regression coefficient) AND variation (e.g. standard deviation) or associated estimates of uncertainty (e.g. confidence intervals) |
| ☐ | ☒ | For null hypothesis testing, the test statistic (e.g. $F$, $t$, $r$) with confidence intervals, effect sizes, degrees of freedom and $P$ value noted<br>*Give P values as exact values whenever suitable.* |
| ☒ | ☐ | For Bayesian analysis, information on the choice of priors and Markov chain Monte Carlo settings |
| ☒ | ☐ | For hierarchical and complex designs, identification of the appropriate level for tests and full reporting of outcomes |
| ☐ | ☒ | Estimates of effect sizes (e.g. Cohen's $d$, Pearson's $r$), indicating how they were calculated |

*Our web collection on statistics for biologists contains articles on many of the points above.*

## Software and code

Policy information about availability of computer code

| | |
|---|---|
| Data collection | Data were obtained from public sources as described in the text. GBIF presence records were obtained with the R package spocc v.1.2.0 (script documentation available at https://ieco-lab.github.io/slfrsk).<br><br>A Code availability statement has been provided as follows:<br><br>All code written and used for this study are available as an R package that is publicly available at: https://github.com/ieco-lab/slfrsk. |
| Data analysis | MaxEnt v3.4.1, R v4.0.2 (packages therein: cleangeo v0.2-4, DHARMa v0.4.1, doParallel v1.0.16, dplyr v1.0.6, ENMTools v1.0.4, ggfortify v0.4.11, ggplot2 v3.3.3, ggrepel v0.9.1, grid v4.0.2, gridExtra v2.3, here v1.0.1, humboldt v0.9.3.120618, lubridate v1.7.10, lycordata v0.2.0, lycormap v0.1.1, magrittr v2.0.1, patchwork v1.1.1, plotly v4.9.3, raster v3.4-10, RColorBrewer v1.1-2, rgdal v1.5-23, rgeos v0.5-5, RStoolbox v0.2.6, scales v1.1.1, spocc v1.2.0, scrubr v0.3.2, sf v0.9-8, stargazer v5.2.2, stringr v1.4.0, tcltk v4.0.2, tigris v1.4, tools v4.0.2, usethis v2.0.1). R analyses are detailed in the research compendium (available at https://ieco-lab.github.io/slfrsk) and corresponding companion R package, slfrsk (available at https://github.com/ieco-lab/slfrsk). |

For manuscripts utilizing custom algorithms or software that are central to the research but not yet described in published literature, software must be made available to editors and reviewers. We strongly encourage code deposition in a community repository (e.g. GitHub). See the Nature Portfolio guidelines for submitting code & software for further information.

## Data

Policy information about availability of data

All manuscripts must include a data availability statement. This statement should provide the following information, where applicable:

- Accession codes, unique identifiers, or web links for publicly available datasets
- A description of any restrictions on data availability
- For clinical datasets or third party data, please ensure that the statement adheres to our policy

All datasets generated during and/or analyzed in this study are available as described in the methods and research compendium (https://ieco-lab.github.io/slfrsk) or can be obtained as a part of the companion R package (https://github.com/ieco-lab/slfrsk). Cumbersome datasets (e.g., SDM files) are available in a Data Dryad repository (https://doi.org/10.5061/dryad.msbcc2g1b).

# Field-specific reporting

Please select the one below that is the best fit for your research. If you are not sure, read the appropriate sections before making your selection.

☐ Life sciences ☐ Behavioural & social sciences ☒ Ecological, evolutionary & environmental sciences

For a reference copy of the document with all sections, see nature.com/documents/nr-reporting-summary-flat.pdf

# Ecological, evolutionary & environmental sciences study design

All studies must disclose on these points even when the disclosure is negative.

| | |
|---|---|
| Study description | We develop the paninvasion severity assessment framework and use it to assess a rapidly spreading regional US grape pest, the spotted lanternfly planthopper (Lycorma delicatula; SLF), to spread from the invaded US region and disrupt the global wine market.<br><br>To calculate SLF paninvasion risk, we estimate three invasion potentials for each country and US state: transport potential as the average annual tonnage of traded commodities with the invaded US region, establishment potential as the maximum suitability from a novel ensemble of three MaxEnt species distribution models, and impact potential as the annual average production of wine and grapes. We calculate the alignment of these potentials as the Spearman rank correlations between impact potential and the predicted values from linear regressions models of each impact potential regressed on transport and establishment potentials (multivariate regression). Global paninvasion risk was ultimately calculated as a rescaled Pearson correlation between predicted invasion risk (predicted country grape production from impact potential as grape production regressed on transport and establishment potentials) to wine export market size for countries. We also produced a county level map of SLF established populations and regulatory incidents (evidence of transportation but no establishment) for the US. |
| Research sample | No specific sampling was conducted, but data sources are as follows:<br><br>Transport potential data spanning 2012–2017 were obtained from the US Freight Analysis Framework for interstate imports and from the US Trade Online database for international imports, both accessed on June 14, 2019.<br><br>Establishment potential data were presence records for SLF and TOH obtained from GBIF on October 20, 2020; potential bioclimatic variables were obtained from WorldClim and Google Earth (see Methods and SI for more specific sources).<br><br>Impact potential data were grape and wine production during 2012–2017: grape production from the Food and Agriculture Organization of the United Nations crop database ( FAOSTAT, countries) and the USDA National Agricultural and Statistics Service commodity database (states), both accessed on January 24, 2020; wine production from FAOSTAT (countries), accessed on June 21, 2019; and wine production from the Alcohol and Tobacco Tax and Trade Bureau (TTB, states), accessed on June 22, 2019.<br><br>Major viticultural regions were obtained from a TTB US state data set and the global viticultural regions Wikipedia list, both accessed on April 22, 2020.<br><br>Wine market size for 223 countries was the value of wine exports corresponding with the years for our trade data (2012–2017) downloaded from the FAOSTAT detailed trade matrix, accessed August 31, 2020. |
| Sampling strategy | Sample sizes were based on the scope of the research questions, available data (e.g., viticulture and trade data published for states and countries), and best practices to avoid spurious relationships (e.g., using all possible GBIF records while avoiding spatial autocorrelation of those records used in species distribution models). |
| Data collection | Data were obtained from existing repositories and databases available to the public as outlined in the methods. Exact details are outlined with sources in the Methods and SI. |
| Timing and spatial scale | Data were collected from publicly available repositories beginning in June 2019 and ending in October 2020. Analyses spanned terrestrial regions of the earth but were largely partitioned into individual countries and US states. Trade and viticulture production data were obtained for 2012–2017 based on data availability and the timing of SLF invasion in the US. Global presence records for SLF and TOH were obtained as of October 2020. Further details are outlined in the Methods and SI. |
| Data exclusions | Establishment potential: Western Sahara, lacked data to use for transport potential |

| | |
|---|---|
| Data exclusions | Alignment analyses: Antarctica, Monaco, Norfolk Island, Spratly Islands (all lacked pixels in the maxent model layers); United States (avoiding duplicating data across analyses for country alignment plot); China, India, Japan, South Korea, Taiwan, Vietnam (to avoid including countries with SLF populations as of 2020 in invasion potential alignment calculations but were still included in visualizations regardless); Akrotiri and Dhekelia, American Samoa, Bouvet Island, British Indian Ocean Territory, Caspian Sea, Christmas Island, Clipperton Island, Cocos Islands, Falkland Islands, Faroe Islands, French Southern Territories, Heard Island and McDonald Islands, Mayotte, Northern Mariana Islands, Paracel Islands, Pitcairn Islands, Saint Pierre and Miquelon, Tokelau, United States Minor Outlying Islands, Wallis and Futuna (trade data not reported for transport potential); Philippines (no wine production data).<br><br>Global paninvasion risk: All countries removed from the alignment analyses. |
| Reproducibility | Not applicable (no experiments were conducted and data and analyses have been made available via research compendium, https://ieco-lab.github.io/slfrsk and corresponding R package, https://github.com/ieco-lab/slfrsk). |
| Randomization | Species distribution models included randomization of presence record partitions used with MaxEnt for crossvalidation. Otherwise, randomization does not apply to the current study, as it focuses on identifying paninvasion risk based on linking data to the identity of states and countries. |
| Blinding | Not applicable (individual geopolitical units, species identification, etc. were essential to interpretation of analyses). |

Did the study involve field work? ☐ Yes ☒ No

# Reporting for specific materials, systems and methods

We require information from authors about some types of materials, experimental systems and methods used in many studies. Here, indicate whether each material, system or method listed is relevant to your study. If you are not sure if a list item applies to your research, read the appropriate section before selecting a response.

## Materials & experimental systems

| n/a | Involved in the study |
|---|---|
| ☒ | ☐ Antibodies |
| ☒ | ☐ Eukaryotic cell lines |
| ☒ | ☐ Palaeontology and archaeology |
| ☒ | ☐ Animals and other organisms |
| ☒ | ☐ Human research participants |
| ☒ | ☐ Clinical data |
| ☒ | ☐ Dual use research of concern |

## Methods

| n/a | Involved in the study |
|---|---|
| ☒ | ☐ ChIP-seq |
| ☒ | ☐ Flow cytometry |
| ☒ | ☐ MRI-based neuroimaging |
